# Birth and death of a protein

**DOI:** 10.7554/eLife.29502

**Published:** 2017-07-20

**Authors:** Julie Clément, Bernard de Massy

**Affiliations:** 1 Institut de Génétique Humaine, CNRS-Université de Montpellier, Montpellier, France; 1 Institut de Génétique Humaine, CNRS-Université de Montpellier, Montpellier, FranceBernard.De-Massy@igh.cnrs.fr

**Keywords:** vertebrates, PRDM9, recombination, hotspot, meiosis, Xiphophorus, Other

## Abstract

The ways in which recombination sites are determined during meiosis are becoming clearer following a phylogenomic analysis for 225 different species.

**Related research article** Baker Z, Schumer M, Haba Y, Bashkirova L, Holland C, Rosenthal GG, Przeworski M. 2017. Repeated losses of PRDM9-directed recombination despite the conservation of PRDM9 across vertebrates. *eLife* **6**:e24133. doi: 10.7554/eLife.24133

Each of our cells carry two almost identical copies of each of our chromosomes, one copy inherited from each parent. These small differences, which are mainly caused by mutations, make an important contribution to the genetic diversity observed in humans and other species. During meiosis, the chromosomes from each parent pair up and then swap segments of DNA: this process, which is known as meiotic recombination, is important for diversity and is essential for fertility (Hunter, 2015). An important scientific goal is to understand where recombination occurs on chromosomes, what molecular processes are involved, and how meiotic recombination affects the evolution of the genome.

Many components of the molecular machinery involved in meiotic recombination are similar in fungi, plants and animals. However, some features of meiotic recombination have not been conserved across species. For example, in all yeast, plant and vertebrate species studied to date, meiotic recombination happens at specific regions within the chromosomes known as hotspots. In flies and worms, on the other hand, it happens at many more locations within the chromosomes. Moreover, there are two main pathways that direct meiotic recombination to hotspots.

The hotspots in yeast, plant and some vertebrate species are located at regions of the genome that are easier to access, such as regulatory regions, transcription start sites or CpG islands (regions with a high frequency of CG dinucleotides; de Massy, 2013). These hotspots are stable over evolutionary time (Lam and Keeney, 2015; Singhal et al., 2015) and this pathway is likely ancestral among eukaryotes.

In contrast, the hotspots in many mammals, including humans, apes and mice, are found at locations where a protein called PRDM9 can bind to the DNA (Figure 1). In particular, the binding sites (and, therefore, the location of the hotspots) are determined by the amino acid sequence of the zinc finger domain of PRDM9 and are characterized by histone modifications mediated by the PR/SET domain of PRDM9 (Baudat et al., 2010; Myers et al., 2010; Parvanov et al., 2010). However, over the course of time the binding sites have rapidly accumulated mutations that can disrupt the PRDM9 binding. This is correlated with the fast evolution of the zinc finger domain, which ensures that PRDM9 can maintain its binding activity in the genome.

**Figure 1.**
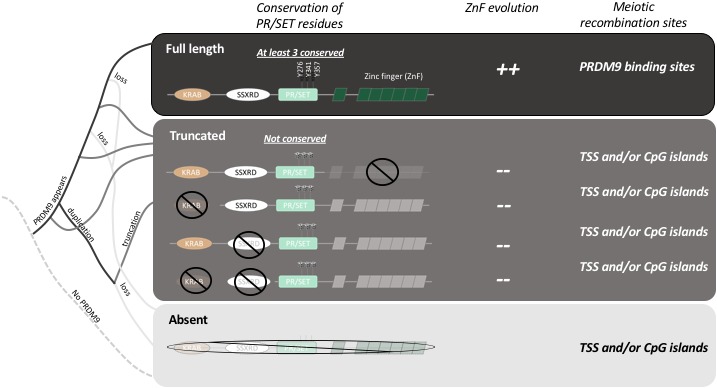
The evolution of PRDM9 and meiotic recombination. The full-length PRDM9 protein contains four domains: KRAB, SSXRD, PR/SET and ZnF (short for Zinc finger). PRDM9 plays an important role in meiotic recombination but, over the course of evolution, many species have either completely lost this protein (light grey line in the simplified phylogenetic tree to the left), or now carry truncated versions of it (dark grey lines). In species with full-length PRDM9 (top row), recombination hotspots are located at sites where PRDM9 binds to the DNA in the genome, and three residues in the PR/SET domain that are essential for the recombination process (Y276, Y341 and Y357; Wu et al., 2013) are conserved: moreover, the locations of the hotspots change over time due to the rapid evolution of the ZnF domain. In species with truncated PRDM9 (next four rows), recombination hotspots are located at transcription start sites (TSS) or at CpG islands, the three residues in the PR/SET domain are not conserved, and the ZnF domain (if present) does not evolve rapidly. In species that lack PRDM9 (bottom row), recombination hotspots are located at transcription start sites (TSS) or at CpG islands.

It is still only partly understood why such a molecular strategy has evolved and why the PRDM9 gene is present in some vertebrates but lost in others (Ponting, 2011). Now, in eLife, Molly Przeworski of Columbia University and colleagues – including Zachary Baker and Molly Schumer as joint first authors – report the results of a series of experiments that shed light on some of these questions (Baker et al., 2017).

By analyzing the DNA and RNA sequences of 225 different species, Baker et al. demonstrate that PRDM9 is not specific to mammals, and they show that even distantly related animals have genes that produce their own equivalents of the PRDM9 protein. They go on to show that while the zinc finger domain evolves rapidly in these species, the other three domains in PRDM9 (the KRAB, SSRXD and PR/SET domains) are conserved (Figure 1). This suggests that the role of PRDM9 appeared very early during the evolution of vertebrates and has been maintained in these different lineages.

Baker et al. – who are based at Columbia, Harvard, Texas A&M and the CICHAZ research station in Mexico – also discovered that several species had independently lost the ability to make PRDM9, while others produced only truncated versions of the protein. For example, the version of PRDM9 found in teleost fish does not contain a domain called the KRAB domain, which is found in full-length PRDM9: moreover, the zinc finger domain is not evolving rapidly in these fish, and three of the amino acids involved in the catalytic activity of PR/SET are not conserved (Figure 1). It appears that the truncated PRDM9 proteins have no influence on the location of recombination hotspots.

Baker et al. investigated this hypothesis by analyzing recombination sites in swordtail fish hybrids between two species of fish belonging to the genus *Xiphophorus*. In these hybrids, in which PRDM9 lacks both the KRAB and SSXRD domains, meiotic recombination occurs preferentially at CpG islands, similar to what has been reported for dogs and birds, which completely lack the gene for PRDM9 (Figure 1; Auton et al., 2013; Axelsson et al., 2012; Singhal et al., 2015). This suggests that the ancestral pathway of recombination (that is, a pathway that existed before the emergence of PRDM9) is active in swordtail fish. Moreover, since the gene for PRDM9 is still under selection in these fish, it must have other roles that remain to be determined. Thus, two important new conclusions emerge from the work of Baker, Schumer, Przeworski and colleagues. First, the full length PRDM9 is required for directing recombination, and second, a shorter version of the protein may still have a relevant role.

Overall, there is also a clear divide between the two hotspot localization pathways. However, several questions remain open, and more research is needed to discover why PRDM9 has evolved in the first place, and why it is maintained in some species but not in others. Answers to these puzzles may come from a better understanding of the underlying molecular properties and how the different pathways have affected the pattern of genetic diversity and selection.
